# Concurrent severe pulmonary tuberculosis with Evans syndrome: a case report with literature review

**DOI:** 10.1186/s12879-022-07512-1

**Published:** 2022-06-13

**Authors:** Xiao-hong Pan, Jie-kun Xu, Lei Pan, Cai-hong Wang, Xiao-qing Huang, Jun-ke Qiu, Xiao-bo Ji, Min-jie Mao

**Affiliations:** Tuberculosis Care Unit, Affiliated Hangzhou Chest Hospital, Zhejiang University School of Medicine, No. 208 Huancheng East Road, Hangzhou, 310003 Zhejiang China

**Keywords:** Tuberculosis, Evans syndrome, Autoimmune hemolytic anemia, Coombs test, Glucocorticoids

## Abstract

**Background:**

Tuberculosis is a bacterial infection involving multiple organs and systems. Its hematological presentation mainly includes anemia and leukocytosis. Evans syndrome is a rare autoimmune disease characterized by autoimmune hemolytic anemia, immune thrombocytopenia, and neutropenia, with positive results for the direct Coombs test and platelet antibodies. The cooccurrence of tuberculosis and Evans syndrome is rarely reported.

**Case presentation:**

A 69-year-old female presented with a fever and shortness of breath. Her chest computerized tomography scan showed extensive miliary nodules in the bilateral lung fields. She rapidly developed respiratory failure that required endotracheal intubation and mechanical ventilation. The acid-fast bacilli sputum smear results indicated a grade of 3+. Later on, blood testing revealed hemolytic anemia, a positive direct Coombs test result, and the presence of the platelet antibody IgG. This patient was diagnosed as having disseminated pulmonary tuberculosis and Evans syndrome. She successfully recovered after treatment with antituberculosis drugs and glucocorticoids.

**Conclusions:**

Tuberculosis can occur together with Evans syndrome. Affected patients should receive both antituberculosis and immunosuppressive drugs.

## Background

Tuberculosis is a contagious bacterial infectious disease caused by *Mycobacterium tuberculosis*. Its main presentation is infection of the respiratory tract, but tuberculosis can be extrapulmonary and involve multiple organs and systems. In patients with tuberculosis, hematological system presentations mainly include anemia and leukocytosis [1], which can occur in many other diseases. Patients with an immunocompromised status have an increased risk of tuberculosis infection [2]. Tuberculosis can also affect the immune system and is related to autoimmune disorders [3]. Evans syndrome is a rare autoimmune disease characterized by autoimmune hemolytic anemia (AIHA), immune thrombocytopenic purpura (ITP), and neutropenia, with positive results for the direct Coombs test and platelet antibodies [4]. Patients with Evans syndrome are usually treated with glucocorticoids. However, glucocorticoids can cause immunosuppression, which increases the risk of tuberculosis infection and makes its management more challenging. Here, we report a patient with no medical history of disease but who was diagnosed as having concurrent severe pulmonary tuberculosis and Evans syndrome. The patient had a satisfactory outcome after our management.

## Case presentation

**Fig. 1:**
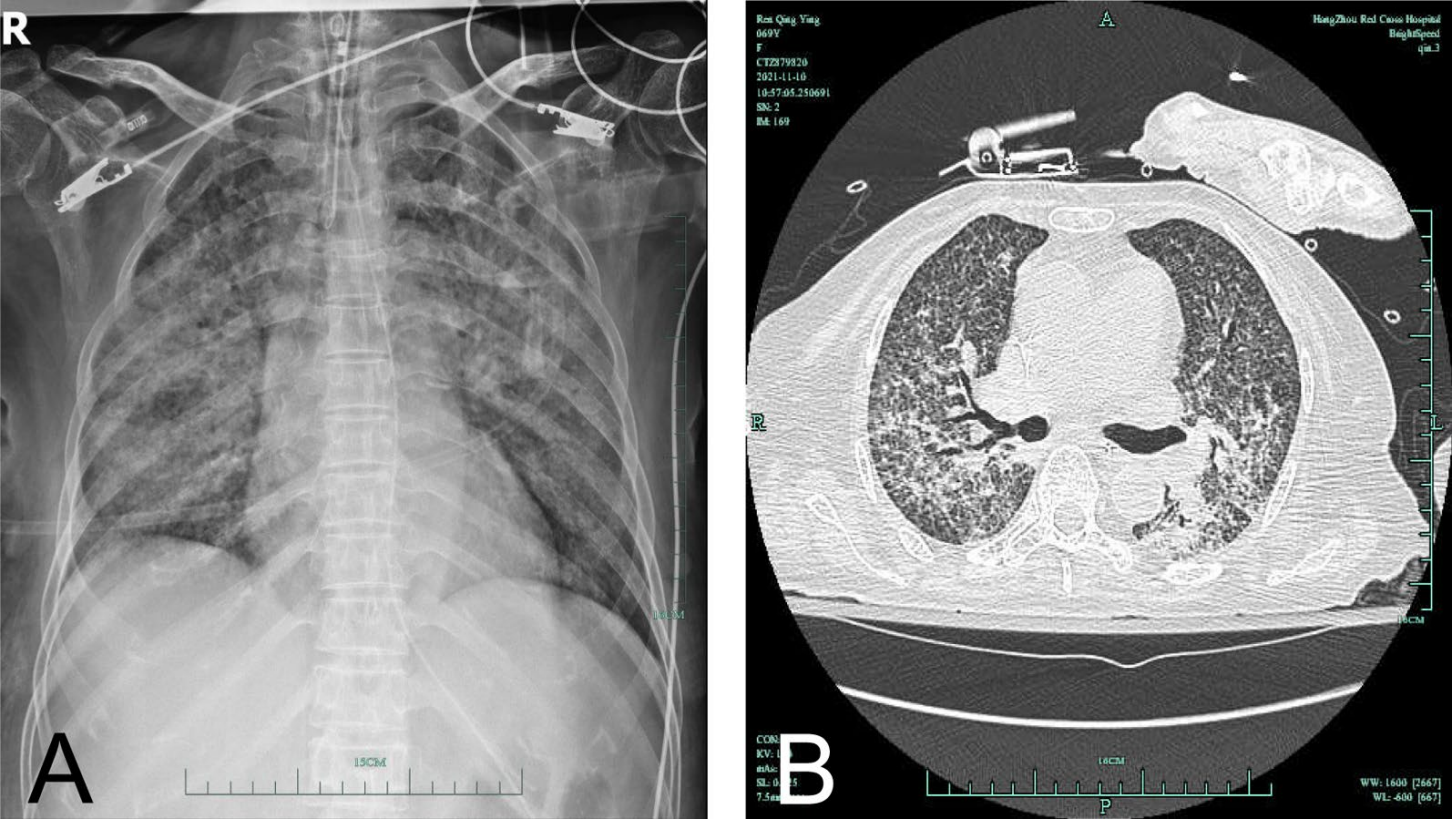
Chest imaging findings during the hospital admission. **A** The X-ray shows increased bilateral lung markings with diffuse patchy infiltration. **B** The computed tomography scan shows bilateral diffuse patchy miliary nodules

A 69-year-old female went to a local hospital due to generalized weakness and a poor appetite for more than 1 month as well as a fever and shortness of breath for 5 days. She received a chest computerized tomography scan, which showed extensive miliary nodules in the bilateral lung fields with an uneven distribution and partial fusion. Her shortness of breath exacerbated, and her oxygen saturation decreased rapidly. She received endotracheal intubation and mechanical ventilation. Later on, her acid-fast bacilli sputum smear results indicated a grade of 3+. For treatment, the patient received isoniazid, rifampicin, pyrazinamide, and ethambutol. On November 1, 2021, she was transferred to our Tuberculosis Care Unit at the Hangzhou Chest Hospital affiliated to Zhejiang University, Zhejiang, China, with a diagnosis of severe pulmonary tuberculosis. During the hospital admission, the physical examination revealed a body temperature of 36.2 °C, a pulse of 95 beats/min, and a blood pressure of 105/40 mmHg (under intravenous norepinephrine, 0.1 μg/kg/min). The patient was intubated orotracheally and looked calm and anemic, with slight conjunctival icterus. There was no sign of bleeding, petechia, or purpura. A few crackles could be heard in the bilateral lung fields. Her abdomen was flat and soft, with no tenderness. The liver and spleen were not palpated. Routine blood testing showed a white blood cell count of 4.9 × 10^9^/L, a hemoglobin concentration of 73 g/L, and a platelet count of 16 × 10^9^/L. The blood coagulation panel showed the following results: prothrombin time, 17.2 s; activated partial thromboplastin time, 44.6 s; d-dimer concentration, 28,800 μg/L; fibrinogen concentration, 109 mg/dL, and international normalized ratio, 1.45. Other laboratory tests showed the following concentrations: serum procalcitonin, 3.91 ng/mL; amylase, 39 U/L; uric acid, 274 μmol/L; total bilirubin, 76.3 μmol/L, cholinesterase, 1122 U/L; creatinine, 229 μmol/L; lactate dehydrogenase, 603 U/L; and albumin, 24.6 g/L. The blood smear results showed < 1% fragmented red blood cells, a normal reticulocyte count, a disintegrin and metalloproteinase with thrombospondin motifs 13 (ADAMTS13) activity of 12.46%, and negative for ADAMTA13 inhibitor antibody. In addition, the patient tested positive for the direct Coombs test and the platelet antibody IgG, as well as negative by the indirect anti-human globulin test. The chest X-ray showed bilateral patchy infiltration, and the computed tomography scan showed bilateral diffuse patchy miliary nodules (Fig. 1). The final diagnosis of the patient included subacute hematogenous disseminated pulmonary tuberculosis, acute respiratory failure, Evans syndrome, renal insufficiency, hepatic failure, thrombocytopenia, disseminated intravascular coagulation, and hypoalbuminemia. Mechanical ventilation was continued after hospital admission. Moreover, the patient received antibiotics including isoniazid (0.3 g, daily), rifapentine (0.45 g, twice a week), and sulbactam and cefoperazone (2.0 g, every 8 h). An intravenous injection of dexamethasone (10 mg, daily) and a subcutaneous injection of recombinant human thrombopoietin (1500 U, daily) were also administered. After this management, the oxygenation of the patient improved. The laboratory test results also showed that the platelet count increased to 63 × 10^9^/L and that the hemoglobin level recovered to 80 g/L. Endotracheal intubation was removed on November 8, 2021. The patient was discharged from the hospital with oral prednisone (15 mg, daily), antituberculosis medications [isoniazid (300 mg, daily), rifampicin (600 mg, daily), and ethambutol (750 mg, daily)], and the antibiotic levofloxacin (500 mg, daily). She was followed up in the clinic in stable condition.

## Discussion and conclusion

Evans syndrome is a rare immune system disease that is characterized by AIHA, ITP, and immune neutropenia [4]. Evans syndrome occurring concurrently with tuberculosis is extremely rare. Depending on the optimal reaction temperature of the antierythrocyte autoantibodies, AIHA is mainly classified into two types: warm AIHA and cold agglutinin syndrome [5]. Patients with Evans syndrome usually have warm AIHA, in which IgG antibodies react with red blood cell surface antigens at a body temperature of ≥ 37 °C. In ITP, the immune system produces antibodies directly against platelet membrane glycoproteins (GPIIb/IIIa) [6]. The etiology of Evans syndrome is currently considered to be primary or secondary, depending on the presence or absence of secondary factors. Secondary Evans syndrome is thought to be due to systemic lupus erythematosus (SLE), autoimmune lymphoid tissue proliferation, non-Hodgkin’s lymphoma, or viral infections (e.g., human immunodeficiency virus, hepatitis C) [7]. The treatment of Evans syndrome is mainly based on the clinical experience of the physicians managing the patient’s AIHA and ITP. Glucocorticoids are the first-line treatment. Prednisone or prednisolone at 1–2 mg/kg/day, with severe cases requiring a dose of 4–6 mg/kg/day over 72 h, can be given to affected patients. Second-line therapy includes intravenous human gamma globulin, rituximab, mycophenolate mofetil, cyclosporine, and cyclophosphamide, which have an overall effective remission rate of about 76% [8]. Relapses of Evans syndrome have been reported in patients who stop taking their medications. Nevertheless, patients with Evans syndrome require immunosuppressive therapy, which increases their risk of infectious diseases such as tuberculosis.

Tuberculosis is a chronic bacterial infectious disease and a notifiable communicable disease in China. Despite its decreasing incidence in China in recent years, a significant number of tuberculosis cases are still reported annually [9]. Standard treatments commonly include three or four antitubercular medications. Patients with an immunocompromised status, such as those with human immunodeficiency virus infection or undergoing glucocorticoid/immunosuppressive therapy, have an increased risk of developing severe tuberculosis. When Evans syndrome occurs at the same time as tuberculosis, treatment can be challenging, since the immunosuppressive therapy required to treat Evans syndrome might exacerbate the tuberculosis infection.

There are only a few previous reports on patients with Evans syndrome and tuberculosis (Table 1). In 1995, Kim et al. reported on a male patient who received treatment for tuberculosis 20 years ago. He was also diagnosed with Evans syndrome 2 years ago and received immunosuppressive therapy [10]. He presented to the hospital due to an expanding perianal ulcer. Finally, tuberculosis was confirmed after biopsy, and the lesion was resolved after antitubercular therapy. The authors considered that the impaired immune system from Evans syndrome and its long-term immunosuppressive therapy may have been risk factors for this patient to have recurrence of tuberculosis.

**Table 1:** Characteristics of the patients in the previous and current case report studies

Article	Patient	Presentation	Laboratory findings	Imaging studies	Bacteriology	Treatment	Outcome
Kim et al. [10]	52-year-old man with a history of Evans syndrome	Expanding perianal ulcer	Hemoglobin: 11.2 g/dL, hematocrit: 34.6%, white blood count: 6900/μL, platelet: 97,000/μL	Chest X-ray: irregular densities and infiltration in both upper lung fields	Sputum and wound smear: acid-fast bacilli. Culture grew *M. tuberculosis*	Isoniazid, rifampin, ethambutol, pyrazinamide	Ulcer healed
Morell et al. [11]	25-year-old woman	Left axillary lymphadenopathy, thrombopenia	Platelet count changed from 86,000/μL to 7000/μL in 2 days, hemoglobin: 12.5 g/dL, hematocrit: 41%, white blood count: 8500/μL, Positive Coombs test after gamma globulin treatment	Normal chest X-ray and chest/abdominal/pelvic CT scan	Lymph node biopsy confirmed acid-fast bacilli and *Mycobacterium tuberculosis*	Gamma globulin, Antitubercular quadritherapy	Recovered
Sharma et al. [12]	30-year-old woman	Fever, weight loss, petechiae, purpura	Hemoglobin: 48 g/dL, platelet: 5000/μL, positive Coombs test	X-ray/CT: infiltration in bilateral middle lung fields, necrotic mediastinal and mesenteric nodes	Sputum smear revealed acid-fast bacilli	Category 1 antitubercular therapy, hydroxychloroquine, methylprednisolone, and then corticosteroids	Recovered
Shi et al. [13]	26-year-old pregnant woman, with a history of Evans syndrome	Cough, fever, dyspnea	Hemoglobin: 101 g/dL, platelet: 73,000/μL, white cell count: 13,860/μL, negative Coombs test	Chest X-ray/CT: diffuse bilateral infiltration and pleural effusion. Abdominal CT splenomegaly	Sputum PCR showed *M. tuberculosis*	Mechanical ventilation, antituberculosis therapy (isoniazid, rifampin, ethambutol, pyrazinamide, moxifloxacin), methylprednisolone, plasma exchange	Fetal demise, patient recovered
Gyawali et al. [14]	20-year-old man	Melena, fever	Hemoglobin: 5.3 g/dL, white blood count: 5400/μL, platelet: 319,000/μL, positive Coombs test	Chest X-ray/CT: multiple centriacinar nodules, right pleural effusion	Sputum negative for acid-fast bacilli	Isoniazid, rifampin, pyrazinamide, ethambutol, prednisone	Increased fatigue, weakness after stopping prednisone, recovered after methylprednisone and cyclosporine
Current case	69-year-old woman	Fever, shortness of breath	Hemoglobin: 7.3 g/dL, white blood count: 4900/μL, platelet: 160,000/μL	Chest X-ray/CT: bilateral diffuse patchy infiltration and miliary nodules	Sputum smear result of acid-fast bacilli: 3+	Isoniazid, rifapentine, sulbactam/cefoperazone	Recovered

*CT* computed tomography

Morell et al. described a patient who presented with left axillary lymphadenopathy [11]. Laboratory test results showed autoimmune hemolytic anemia and thrombocytopenia, with positive antiplatelet antibody and Coombs test results. The patient received gamma globulin treatment, which improved the platelet count. However, autoimmune hemolytic anemia persisted. A further lymph node biopsy confirmed tuberculosis. The patient’s anemia eventually improved after antitubercular quadritherapy. The authors suggested that tuberculosis could induce hematological changes, including hemolytic anemia and thrombocytopenia. In patients with Evans syndrome and poor responses to immunosuppressive therapy, other causes, including tuberculosis, should be ruled out.

Sharma et al. presented a case of disseminated tuberculosis combined with SLE and Evans syndrome [12]. The patient presented with a fever, gum bleeding, epistaxis, and skin purpura lesions. She had anemia, thrombocytopenia, and a positive Coombs test result. Chest imaging showed bilateral pulmonary consolidation with necrotic mediastinal and mesenteric lymphadenopathy, and she tested positive for antinuclear antibody. Therefore, the patient was diagnosed with disseminated tuberculosis and Evans syndrome. Immunosuppressive therapy did not improve her hematological findings, which were only corrected after antitubercular therapy was added. The authors concluded that Evans syndrome was secondary to disseminated tuberculosis or immune disorders of SLE.

Shi et al. described a pregnant woman with a previous history of Evans syndrome who presented with a fever and pneumonia [13]. After confirming the diagnosis of tuberculosis based on a positive test of *Mycobacterium tuberculosis* in the sputum and bronchoalveolar lavage fluid, antitubercular therapy and methylprednisolone were administered. However, the patient’s condition deteriorated and rapidly developed into acute respiratory distress syndrome. Laboratory test results indicated that the patient had pancytopenia. Disseminated tuberculosis-associated hemophagocytic lymphohistiocytosis was considered. The patient survived after receiving antitubercular medications, high-dose methylprednisolone, intravenous immunoglobulin, and plasma exchange therapy. The authors concluded that patients with a history of autoimmune diseases, such as Evans syndrome, may be at a high risk of having a life-threatening hyperinflammatory syndrome with tuberculosis infection. Prompt antitubercular therapy with corticosteroids and immunoregulators should be initiated as early as possible.

Gyawali et al. reported on a patient who presented with melena and fever [14]. He was diagnosed with pulmonary tuberculosis combined with hemolytic anemia after a chest imaging study, sputum acid-fast staining, and laboratory testing. Antitubercular medications and prednisone were prescribed. After discontinuation of prednisone, the patient developed significant thrombocytopenia along with severe anemia. Evans syndrome was then considered, which was alleviated by the addition of cyclosporine to the antitubercular therapy.

Overall, in most of these patients, their tuberculosis infections were diagnosed when *Mycobacterium tuberculosis* was identified in the sputum smear or culture when looking for acid-fast bacilli staining or by polymerase chain reaction. Some tuberculosis cases were also diagnosed based on positive *Mycobacterium tuberculosis* results in samples from a lymph node biopsy or bronchoalveolar lavage fluid. Evans syndrome was diagnosed when there was evidence of AIHA and thrombocytopenia. Positive results for the direct Coombs test or antiplatelet antibody test could be used when available to support the diagnosis. All of these patients received antitubercular therapy and immunosuppressive drugs. Their tuberculosis and hematological changes were improved after the combination therapy.

In conclusion, tuberculosis can cause hematological changes. When there is evidence of hemolytic anemia and thrombocytopenia, appropriate tests, such as the direct Coombs test and antiplatelet antibody test, should be performed. Once concurrent tuberculosis and Evans syndrome are confirmed, patients should receive both antitubercular therapy and immunosuppressive drugs. Their clinical courses should be monitored closely to ensure a satisfactory recovery. Long-term therapy still requires further investigation, since immunosuppressive agents for Evans syndrome treatment might increase the risk of tuberculosis infection and recurrence. Close clinical follow-up and repeat evaluations should be offered to these patients.

## Data Availability

Not applicable.

## Abbreviations

AIHA: Autoimmune hemolytic anemia
ITP: Immune thrombocytopenic purpura
ADAMTS13: A disintegrin and metalloproteinase with thrombospondin motifs 13
SLE: Systemic lupus erythematosus
